# Identification of a SCN5A Genetic Variant Associated With Type 1 Brugada Syndrome (BrS) in a Family

**DOI:** 10.7759/cureus.64883

**Published:** 2024-07-19

**Authors:** Jack Jnani, Dorota Gruber, Tafadzwa Mtisi, Moussa Saleh, Bani M Azari

**Affiliations:** 1 Internal Medicine, North Shore University Hospital, Manhasset, USA; 2 Pediatrics and Cardiology, Zucker School of Medicine at Hofstra/Northwell, Manhasset, USA; 3 Cardiology, North Shore University Hospital, Manhasset, USA; 4 Cardiovascular Institute, Northwell Health, New Hyde Park, USA

**Keywords:** brugada syndrome, cardiac arrhythmia, genetic screening, implantable cardiac defibrillator (icd), scn5a mutation

## Abstract

The Brugada pattern is associated with a genetic disorder characterized by ST-segment elevation in the right precordial leads on electrocardiogram (EKG) in the absence of structural heart disease. Patients with the Brugada pattern have an increased risk for ventricular tachyarrhythmia and sudden cardiac death. Loss-of-function mutations in the SCN5A gene which encodes the alpha subunit of the cardiac sodium channel have been associated with Brugada syndrome (BrS). We report a case of a patient who was found to have a spontaneous type 1 Brugada pattern on a routine EKG done prior to travel. He underwent electrophysiological testing (EPS) which provoked ventricular tachycardia and underwent implantable cardioverter defibrillator (ICD) placement. His family history revealed a history of sudden cardiac death, abnormal EKG, syncope, dilated cardiomyopathy, and BrS. Genetic testing revealed a variant of uncertain significance (VUS) in the SCN5A gene in the proband and six of his relatives. The SCN5A VUS in this clinical context and segregation with the disease in his family supports its reclassification to pathogenic.

## Introduction

The Brugada pattern is associated with a genetic disorder characterized by ST-segment elevation on electrocardiogram (EKG) in the absence of structural heart disease, resulting in an increased risk for syncope, ventricular tachyarrhythmia, and sudden cardiac death (SCD). The prevalence is 1:2000, and it is more common among young and middle-aged males [1,2]. The youngest patient clinically diagnosed with the syndrome is two days old and the oldest is 84 years old. The average age at diagnosis is 42 years old [3]. The syndrome is estimated to be responsible for at least 4% of all sudden deaths and at least 20% of sudden deaths in patients with structurally normal hearts [4].

SCN5A variants are associated with several arrhythmia and cardiomyopathy phenotypes, including Brugada syndrome (BrS). Other genes implicated in BrS include SCN1B, SCN3B, CACNA1C, CACNB2, CACNA2D1, KCNE3, KCNJ8, and KCND3 [5]. SCN5A-encoded cardiac sodium channel loss-of-function variants have been shown to confer the pathogenic basis for an estimated 15-30% of BrS [2]. Gain-of-function mutations in SCN5A lead to long QT syndrome type 3 (LQT3), increasing the risk of sudden cardiac events [5]. Pathogenic variants in SCN5A can also contribute to dilated cardiomyopathy, sick sinus syndrome, and sudden infant death syndrome (SIDS) [5]. These mutations can also affect neuronal function possibly explaining some cases of sudden unexpected death in epilepsy (SUDEP) [6]. Pathogenic variants in SCN5A are typically inherited in an autosomal dominant pattern but can exhibit reduced penetrance and variable expressivity, both within and between families [2].

We report a case of a patient with BrS carrying a variant of uncertain significance (VUS) in the SCN5A gene.

This article was previously presented as a meeting abstract at the 2024 American College of Cardiology (ACC) meeting on April 7, 2024.

## Case presentation

The proband is a 24-year-old male who was found to have a spontaneous type 1 Brugada pattern on routine EKG taken for medical optimization prior to travel. He denied a personal history of syncope, chest pain, or palpitations. Family history was significant for SCD, ventricular tachycardia (VT), and BrS.

The patient underwent genetic testing that identified a VUS p.(Leu917Arg) (CTG>CGG): c.2750 T>G in exon 16 of the SCN5A gene.

**Figure 1:**
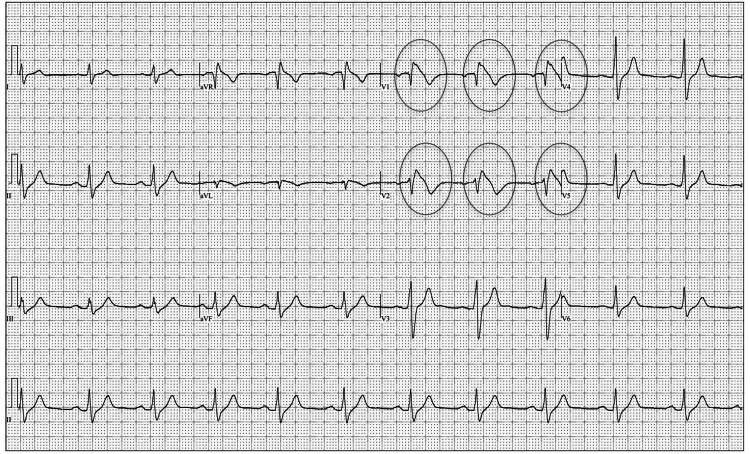
EKG showing type 1 Brugada pattern in the proband in the right precordial leads. The ovals highlight leads V1 and V2 where there are coved ST-segment elevations followed by T-wave inversions characteristic of a type 1 Brugada EKG pattern. EKG: electrocardiogram

Clinically, the patient met electrocardiographic criteria for a type 1 Brugada pattern (Figure 1), and family history was suggestive of genetic channelopathy. Electrophysiological (EP) testing was able to provoke VT, and he underwent an implantable cardioverter defibrillator (ICD) placement. He was also advised to have prompt fever treatment with antipyretics and to avoid drugs that can exacerbate BrS.

Decision-making

Genetic testing identified a VUS in SCN5A. This genetic mutation had previously been described in just one patient as a VUS, and specific clinical information was not provided [7].

A combination approach, both clinical and genetic evaluation, was pursued by him and his family members. 

**Figure 2:**
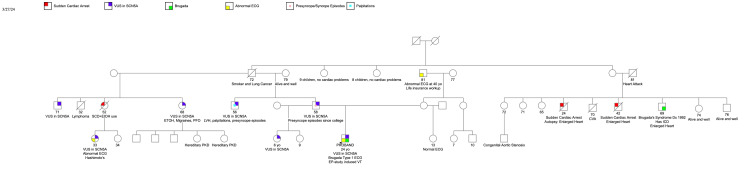
Pedigree analysis of affected family members and their genotype status.

**Figure 3:**
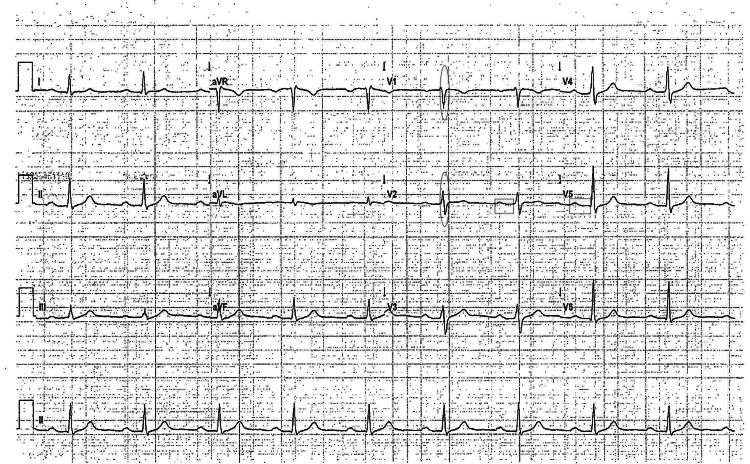
EKG of the proband's paternal uncle (with the SCN5A genetic variant) which is significant for sinus bradycardia, a first-degree AVB, and an IVCD greater than 110 ms. Ovals are drawn over the QRS complexes in selected leads, while rectangles are drawn over the PR intervals in selected leads. EKG: electrocardiogram; AVB: atrioventricular block; IVCD: intraventricular conduction delay

**Figure 4:**
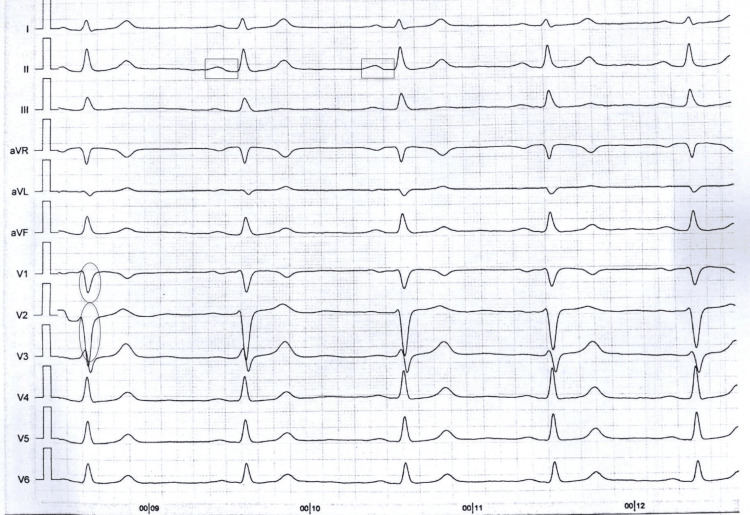
EKG of the proband's paternal half-aunt's daughter (with the SCN5A genetic variant) which is significant for sinus bradycardia, a first-degree AVB, and an IVCD of 113 ms. Ovals are drawn over the QRS intervals in selected leads, while rectangles are drawn over the PR intervals in selected leads. EKG: electrocardiogram; AVB: atrioventricular block; IVCD: intraventricular conduction delay

The VUS was identified in six other family members as seen in the pedigree analysis (Figure 2). His father and his paternal uncle had a history of symptomatic palpitations and frequent presyncope in their 20s that resolved in their late 20s. His paternal uncle had an EKG that was significant for sinus bradycardia, a first-degree atrioventricular block (AVB), and an intraventricular conduction delay (IVCD) greater than 110 milliseconds (ms) (Figure 3). His paternal half-aunt had a history of SCD at the age of 52; her daughter was also found to carry the variant and had a history of palpitations and an EKG with sinus bradycardia and an IVCD of 113 ms (Figure 4). His half-sister who was found to have the SCN5A genetic variant had a type 1 Brugada EKG when she had a fever. More distant family history identified members with a history of SCD and a member with BrS who declined genetic testing. Clinical cardiac evaluation was recommended for all first-degree relatives of the patient regardless of the genotype, pending formal variant reclassification.

Variant evaluation

The SCN5A p.(Leu917Arg) (CTG>CGG): c.2750 T>G variant occurs in exon 16. This missense variant is located in the DII-S6 region of the SCN5A protein, a critical segment that constitutes the central pore-forming region of the sodium channel, determining its ion selectivity. Four other missense variants associated with BrS have been identified in this region, underscoring its importance in the channel's function and its role in the pathogenesis of BrS [8].

Using genomic DNA from the submitted specimen, the relevant portion of the SCN5A gene was PCR amplified, and capillary sequencing was performed. The bi-directional sequence was assembled and aligned to reference gene sequences based on the human genome build GRCh37/UCSC hg19. Sequence alterations were reported according to the Human Genome Variation Society (HGVS) nomenclature guidelines. This method, employed by GeneDx, is expected to have greater than 99% sensitivity in detecting identifiable variants.

The variant's significance was further analyzed using the VarSome genetic database [9], Sorting Intolerant From Tolerant (SIFT) [10], and PolyPhen-2 (Polymorphism Phenotyping v2) [10] to determine the pathogenicity of the SCN5A variant observed in the family. VarSome integrates data from various sources to provide comprehensive variant interpretation. For the SCN5A p.(Leu917Arg) variant, VarSome reported a pathogenic effect in 14 out of 26 individual predictions and classified it as likely pathogenic with a score of 6 points. VarSome uses the American College of Medical Genetics and Genomics (ACMG)/American Association of Molecular Pathology (AMP) guidelines for variant classification, considering criteria such as population frequency, computational and predictive data, functional data, and segregation data. SIFT predicts whether an amino acid substitution affects protein function based on sequence homology and the physical properties of amino acids. For the SCN5A variant, SIFT indicated a damaging effect. 

PolyPhen-2 [10] evaluates the potential impact of an amino acid substitution on the structure and function of a protein using multiple sequence alignments and structural information. The SCN5A variant received a PolyPhen-2 score of 0.923, categorized as "probably damaging" with a sensitivity of 0.68 and a specificity of 0.91. Scores close to 1 indicate a high likelihood of a damaging effect.

These comprehensive bioinformatic analyses collectively suggest that the SCN5A p.(Leu917Arg) variant has a significant deleterious effect on the SCN5A protein, reinforcing its potential involvement in BrS. These bioinformatic analyses are critical in guiding clinical decisions and understanding the genetic basis of the patient's condition.

## Discussion

We report the NM_198056.2 c.2750 T>G p.(L917R) heterozygous variant in the SCN5A gene, together with its segregation in a large family to be associated with BrS, symptomatic palpitations, presyncope, conduction delay, and SCD. The pathogenicity of this variant is supported by its rarity in the general population, multiple pathogenic in silico predictions, and segregation with arrhythmic disease in this family. This variant was previously reported in association with BrS [7]; however, specific clinical information was not provided. Our patient was diagnosed with BrS and received an ICD.

BrS is a genetic disorder that can be potentially life-threatening resulting in an increased risk for VT and sudden cardiac arrest.

Previously, the diagnosis of BrS was solely based on having type 1 EKG changes (spontaneous or induced). However, recently, the Shanghai score, which takes into account clinical history, family history, and genetic history, is used to support the diagnosis. Supporting EKG findings for diagnosis include a spontaneous type 1 EKG or type 2/3 EKG that converts to type 1 after sodium channel blocker (SCB) provocation. Supporting clinical history includes SCD, VT, ventricular fibrillation, and suspected arrhythmic-related syncope. Additionally, supportive family history includes a first- or second-degree relative with BrS, suspicious SCD, or unexplained SCD in a relative less than 45 years of age with a negative autopsy. A probable/definite diagnosis of BrS correlates with Shanghai scores ≥3.5 points, a possible BrS diagnosis relates to a score of 2-3 points, and a non-diagnostic BrS Shanghai score is less than 2 points [4].

A drug challenge for diagnosing BrS is indicated in cases where the disease is suspected but the basal EKG is non-diagnostic. Flecainide, procainamide, disopyramide, and propafenone can be used. A drug challenge is only considered positive if conversion to a type 1 EKG pattern is observed [11].

Given our current knowledge, identifying a specific mutation does not independently make a diagnosis or determine prognosis. However, genetic testing is recommended to support the diagnosis (Shanghai score 0.5 points), to identify relatives who may be at risk, and to further our knowledge of the genotype-phenotype relationship [3]. Current guidelines recommend screening all first-degree relatives of patients diagnosed with BrS or those with otherwise unexplained SCD [12].

Aside from resuscitated cardiac arrest, the clinical factors that have the greatest impact on sudden arrhythmic events (SAEs) in patients with BrS are a history of cardiogenic syncope and the presence of a spontaneous type 1 EKG. In a systematic review of studies involving 4,099 patients, Rattanawong et al. found that patients with spontaneous type 1 EKG had a 2.4% annual incidence of SAEs compared with 0.65% for those with SCB-induced BrS [13]. Males showed a tendency to develop more arrhythmic events than women and have a worse prognosis [11]. The value of inducibility of sustained ventricular arrhythmias during EP study as a risk predictor tool for cardiac events in BrS is controversial based on the available literature [11]. However, a recent prospective study involving 1149 asymptomatic patients from two Italian centers with either spontaneous or drug-induced type 1 EKG pattern demonstrated a relatively low arrhythmic risk in the drug-induced cohort but an increased arrhythmic risk in the spontaneous type 1 EKG subgroup with a positive EP study, suggesting the utility of an EP study in the latter cohort [14]. The presence of early familial SCD was associated with SAEs [15].

BrS patients who also have the SCN5A mutations may have an increased arrhythmogenic risk. For example, in a Japanese study of 415 probands diagnosed with BrS, patients with SCN5A mutations had a higher risk for cardiac events than those without the mutation [16]. A meta-analysis of 1892 BrS patients who underwent SCN5A gene tests revealed that patients with BrS with symptoms at diagnosis and a SCN5A gene mutation were at higher risk of arrhythmic events than those without the mutation [17]. In another meta-analysis of 1780 BrS patients, those with SCN5A mutations exhibited symptoms at a younger age and had a higher rate of type 1 EKG pattern. Those with SCN5A mutations were also at increased risk for major arrhythmic events in the Asian and Caucasian populations [18].

Treatment of patients with BrS includes conservative measures such as prompt treatment of fever with antipyretics and avoidance of drugs that can provoke arrhythmogenic events including SCBs, psychotropic agents, certain anesthetic agents, antihistamines, alcohol intoxication, and illicit cocaine or cannabis use [12].

In patients with BrS who experience electrical storm, intravenous isoproterenol is recommended [4]. Quinidine has been shown to prevent the induction of ventricular fibrillation and suppress spontaneous ventricular arrhythmias in a clinical setting [4]. Quinidine therapy could be considered in patients with ICD and multiple shocks, in cases in which ICD is contraindicated, or for the treatment of supraventricular arrhythmias. It can also be useful in children as a bridge to ICD or an alternative. However, the current literature is limited on its effectiveness, and randomized studies have not been performed [4].

The most effective therapeutic strategy for the prevention of SCD in BrS patients is the ICD [11]. Symptomatic patients displaying the type 1 Brugada EKG (either spontaneously or after sodium channel blockade) who present with aborted sudden death should receive an ICD (class I recommendation). Symptomatic patients presenting with syncope, seizure, or nocturnal agonal respiration also should undergo ICD implantation if deemed to be of cardiac etiology (class I recommendation) [12]. Patients with a spontaneous type I EKG may be considered for an EP study. Asymptomatic patients displaying a type 1 Brugada EKG (either spontaneously or after SCB) should undergo EPS if a family history of SCD is suspected to be from BrS. If inducible ventricular arrhythmia is observed, then a patient should receive an ICD (class IIa recommendation). Asymptomatic patients who have no family history but develop a type 1 EKG after SCB should be closely followed up [4,11].

Radiofrequency (RF) ablation of the arrhythmogenic substrate in the right ventricular outflow tract is another therapy but with limited evidence currently. RF ablation is usually reserved for patients with recurrent ICD shocks that cannot be managed with medical therapy or in those where an ICD is indicated but not implanted (such as those with a strong patient preference to not have an ICD) [12].

## Conclusions

BrS is diagnosed when a type 1 EKG pattern is observed in conjunction with relevant family and clinical history. Patients with BrS are at higher risk of sudden cardiac arrest and should be managed closely. Genetic testing may help support the diagnosis. Treatment may involve conservative management, medical therapy, or ICD placement depending on the select patient.

We identified seven cases of a VUS in the SCN5A gene, c.2750 T>G p.(L917R) in this family, each with a history of symptomatic palpitations or an abnormal EKG. His family history revealed a history of SCD, abnormal EKG, syncope, dilated cardiomyopathy, and BrS in his relatives. Given the clinical, genetic, and family history, we argue that the VUS in the SCN5A gene identified in this patient with BrS is trending towards pathogenicity. Future functional studies to evaluate the mechanism of this variant are warranted. Clinical management in patients with suspicious VUS should include a combination of clinical cardiac evaluation and genetic testing.
